# Supplementary material for: Role of the prefrontal cortex in musical and verbal short-term memory: A functional near-infrared spectroscopy study
Source: Imaging Neurosci (Camb). 2024 May 8;2:imag-2-00168. doi: 10.1162/imag_a_00168 (PMC12272266; doi:10.1162/imag_a_00168)
Supplement: Supplementary Material [file imag_a_00168-supp.pdf]

Role of the prefrontal cortex in musical and verbal short-term memory: A functional near-infrared spectroscopy study

Running title: fNIRS in musical and verbal short-term memory

Jérémie Ginzburg, Anne Cheylus, Elise Collard, Laura Ferreri, Barbara Tillmann, Annie Moulin, Anne Caclin

Supplemental material

fNIRS montage: channel specificity

Hemisphere	Source	Detector	Cortical structure	Specificity (%)
Left	AF7	F5	IFG (p. Triangularis)	54.20
			MFG	25.02
	F7	F5	IFG (p. Triangularis)	82.28
			IFG (p. Orbitalis)	11.66
	FC5	F5	IFG (p. Triangularis)	68.70
			IFG (p. Opercularis)	19.56
	AF3	F5	MFG	66.90
			IFG (p. Triangularis)	22.08
	F3	F1	MFG	68.06
			SFG	30.84
	F3	F5	MFG	60.23
			IFG (p. Triangularis)	38.27
	F3	FC3	MFG	81.08
			PG	9.36
	AF3	AFz	SFG	46.49
			SFG, medial	37.44
	Fz	F1	SFG, medial	40.89
			SFG	39.90
	FC5	FC3	Precentral Gyrus	46.40
			IFG (p. Opercularis)	18.71
Right	AF8	F6	IFG (p. Triangularis)	38.12
			MFG	37.98
	F8	F6	IFG (p. Triangularis)	73.88
			IFG (p. Orbitalis)	18.76
	FC6	F6	IFG (p. Triangularis)	59.66
			IFG (p. Opercularis)	25.22
	AF4	F6	MFG	69.69
			SFG	14.98
	F4	F2	MFG	65.21
			SFG	31.75
	F4	F6	MFG	59.39

			IFG (p. Triangularis)	39.41
	F4	FC4	MFG	70.58
			IFG (p. Opercularis)	10.29
	AF4	AFz	SFG	42.17
			SFG, medial	36.66
	Fz	F2	SFG, medial	40.68
			SFG	35.24
	FC6	FC4	Precentral Gyrus	45.52
			IFG (p. Opercularis)	30.53
Interhemispheric sulcus	Fz	AFz	SFG, medial	43.16
	Fz	FCz	SFG, medial	23.98

Table S1: Each of the 22 recording channels is composed of a source and a detector (located in standard 10-20 positions). We report in the table the two cortical structures for which each channel has the highest specificity (in percent) according to the fOLD software (Zimeo Morais et al., 2018). SFG: Superior Frontal Gyrus, MFG: Middle Frontal Gyrus, IFG: Inferior Frontal Gyrus. The channels forming the IFG, the dlPFC (corresponding to MFG), and the SFG ROIs are highlighted with light blue, light gold, and grey colors respectively. Channels unused in the ROI analysis are not highlighted.

Experiment 1

Behavioral results: criterion

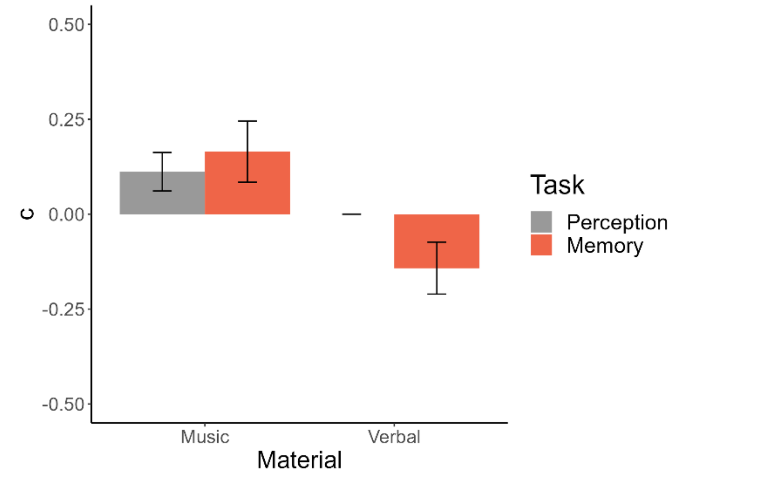

Figure S1: Mean and standard error of criterion (c) as a function of the task (perception in grey/memory in orange) and material (music/verbal).

For the criterion (c), the best model explaining the data included only the material factor (strong evidence, BF_{10} = 89.2) with a more conservative criterion for the musical material than

for the verbal material. One-sample Bayesian t-tests revealed weak evidence for a difference compared to 0 for the perception and memory tasks in the musical material and for the memory task in the verbal material ($1.3 < BF_{10} < 1.7$). Note that for the perception task in the verbal material, all participants displayed a criterion of 0 (no bias) because they performed correctly for all materials.

Topographic representation of HbO results

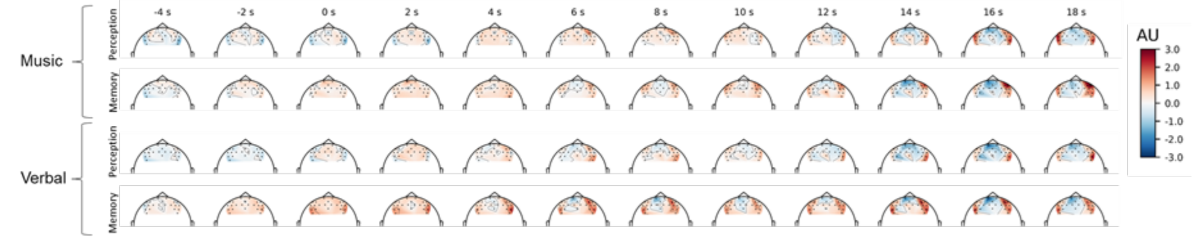

Figure S2: time-course of the topographic representation of the deconvoluted HbO fNIRS signal. Averaged beta across participants are represented every two seconds from -4 to 18 seconds around S1 onset for each channel, each task (perception/memory) and each material (music/verbal). AU: arbitrary units.

HbR results within targeted ROIs

HbR results within the targeted ROIs are summarized in Figure S2.

For the left IFG, we found weak evidence at -5 and -4 seconds before S1 onset for the model including the material effect ($1 < BF_{10} < 1.6$) higher betas for the verbal material as compared to the musical material.

For the right IFG, we found weak evidence -5 seconds before S1 onset for the model including the material effect ($BF_{10} = 2.3$) with higher betas for the verbal material as compared to the musical material. We found weak to decisive evidence -1 to 6 seconds around S1 onset for the model including the task effect ($1.4 < BF_{10} < 160.1$) with lower betas for the memory task as compared to the perception task.

For the left dlPFC, we found weak to strong evidence -5 to 3 seconds around S1 onset for the model including the task effect ($1.1 < BF_{10} < 28.2$) with lower betas for the memory task as compared to the perception task.

For the right dlPFC, we found weak to strong evidence -2 to 2 seconds and 9 to 17 seconds around S1 onset for the model including the task effect ($1.3 < BF_{10} < 13.7$) with lower betas for the memory task as compared to the perception task.

For the SFG, we found weak to positive evidence -2 to 1 seconds around S1 onset for the model including the task effect ($1.6 < BF_{10} < 3.3$) with lower betas for the memory task as compared to the perception task.

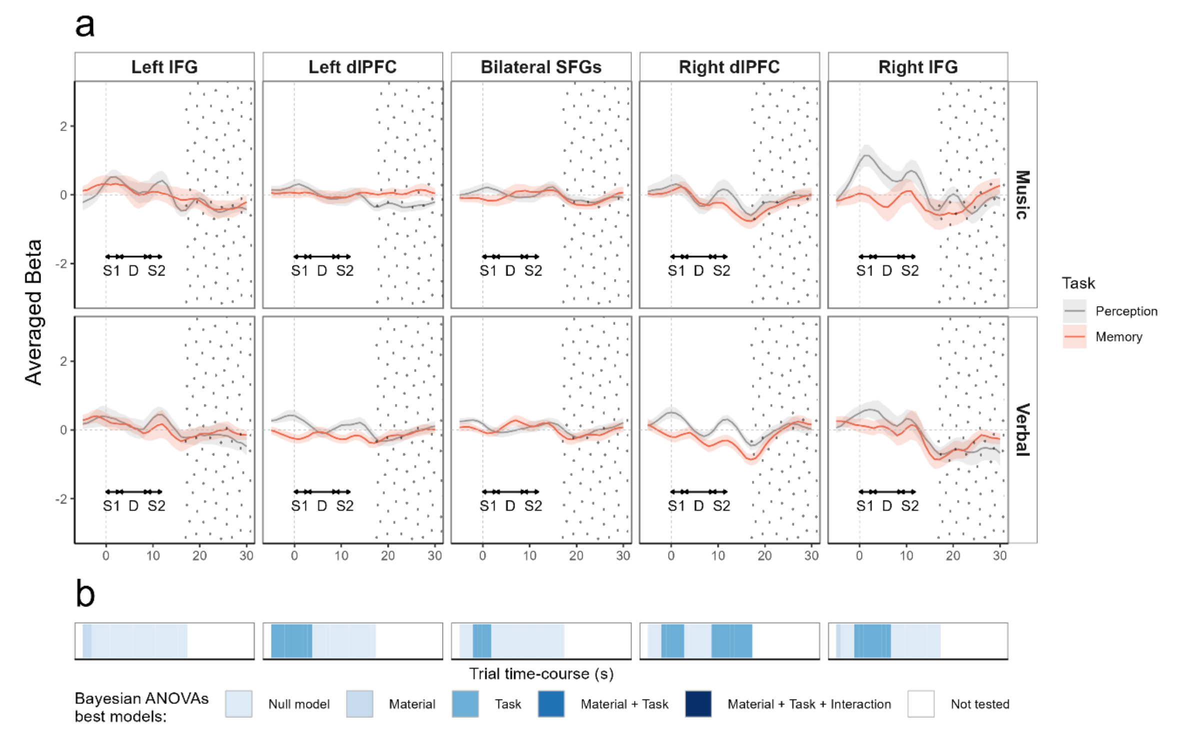

Figure S3: (a) Average beta (plain line) and standard error (shaded area) from the FIR deconvolution performed on HbR data for all participants (n=16), across five ROIs (left and right IFG, left and right dlPFC, bilateral SFGs), in a time window ranging from -5 to 30 seconds around S1 onset (grey dotted vertical line), for the perception task (grey) and the memory task (red). Top panel: musical material; bottom panel: verbal material. For clarity purposes, S1, delay (D), and S2 durations are indicated with double-headed arrows. (b) Time-course representation of Bayesian analysis, each blue shade represents the best model as compared to the null model ($BF_{10} > 1$). No statistics were performed after 18 seconds (end of S2 + peak of the hemodynamic response, ~ 6s), because the deconvoluted signal would then include motor responses which we did not intend to analyze. Dotted areas in all panels represent portions of the signal that were not statistically analyzed.

Experiment 2

Behavioral results: criterion

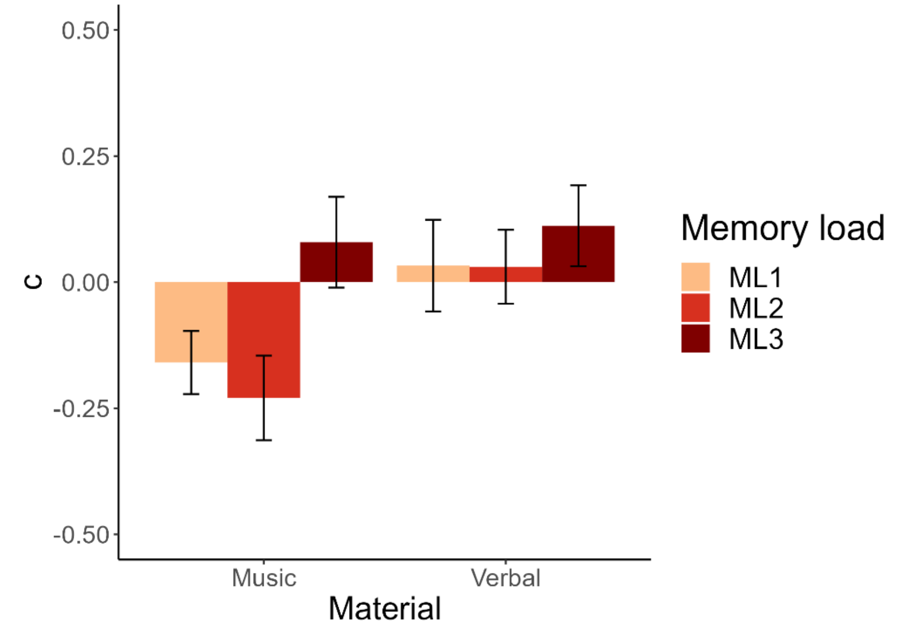

Figure S4: Mean and standard error of criterion (c) as a function of memory load (ML1 in orange/ML2 in red/ML3 in dark red) and material (music/verbal).

For the criterion (c), the best model explaining the data included both the condition and the material factor (positive evidence, $BF_{10} = 6.3$). The analysis of effects across matched models revealed weak evidence for the condition effect ($BF_{inclusion} = 1.72$) and positive evidence for the material effect ($BF_{inclusion} = 3.5$). For the material effect, participants displayed a more conservative criterion for the verbal material as compared to the musical material. Post-hoc Bayesian t-tests for the memory load factor averaged across materials revealed positive evidence for the null model between ML1 and ML2 ($BF_{10} = 0.24$), weak evidence for a difference between ML1 and ML3 ($BF_{10} = 2.5$) with a more conservative criterion for ML3 than for ML1, and positive evidence for a difference between ML2 and ML3 ($BF_{10} = 8$) with a more conservative criterion for ML3 than for ML2. One-sample Bayesian t-tests revealed weak evidence for a difference compared to 0 revealed positive evidence for a difference against for ML1 and ML2 conditions in the musical material ($3 < BF_{10} < 4.2$). For all other comparisons, weak to positive evidence for the null model (no difference compared to 0) was found ($0.2 < BF_{10} < 0.5$).

Topographic representation of HbO results

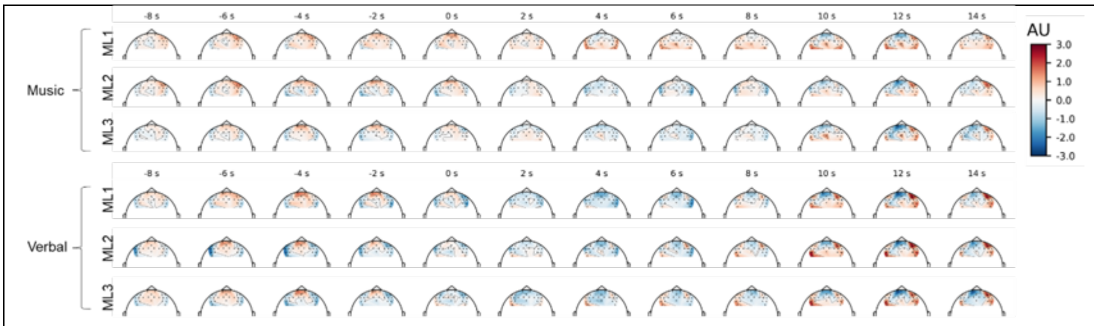

Figure S5: Time-course of the topographic representation of the HbO deconvoluted fNIRS signal. Averaged beta across participants are represented every two seconds from -8 to 14 seconds around delay onset (time sample 0) for each channel, each memory load (ML1/ML2/ML3), and each material (music/verbal). AU: arbitrary units.

HbR results within targeted ROIs

For the left IFG we found weak evidence at -8 seconds before delay onset for the model including the material effect ($BF_{10} = 1.3$) with lower betas for the musical material as compared to the verbal material. We found also weak to positive evidence at 3 and 4 seconds and 10 to 12 seconds after delay onset for the model including the material effect ($1.1 < BF_{10} < 4.7$) with lower betas for the verbal material as compared to the musical material.

For the right IFG we found weak evidence at -9 and -8 seconds before delay onset for the model including the material effect ($1 < BF_{10} < 1.4$) with lower betas for the verbal material as compared to the musical material. We found weak to strong evidence -2 to 4 seconds and 7 to 12 seconds around delay onset for the model including the memory load effect ($1 < BF_{10} < 28.6$). Post-hoc tests averaged over materials for the memory load effect (not shown in Figure S6) revealed weak to positive evidence 0 to 4 seconds after delay onset1 for a difference between ML1 and ML2 ($1.4 < BF_{10} < 7.7$) with lower betas for the ML2 condition as compared to the ML1 one. We found weak evidence 7 to 12 seconds after delay onset for a difference between ML1 and ML3 ($1.5 < BF_{10} < 2.8$) with lower betas for the ML3 condition as compared to ML1 condition. Finally, we found weak to positive evidence for all tested time samples (-2 to 4 and 7 to 12 seconds) for a difference between ML2 and ML3 ($2.1 < BF_{10} < 5.9$) with lower betas for the ML2 condition as compared to the ML3 condition.

For the left dlPFC, we found weak to positive evidence -2 to 2 seconds around delay onset for the model including the material effect ($1.5 < BF_{10} < 8$) with lower betas for the musical material as compared to the verbal material.

For the right dlPFC, we found weak to strong evidence at -9 seconds, -4 to 0 seconds and 10 to 12 seconds around delay onset for the model including the material effect ($1 < BF_{10} < 49.6$) with lower betas for the musical material -9 seconds and -4 to 0 seconds as compared to the verbal material and lower betas for the verbal material 10 to 12 seconds after delay onset as compared to the musical material. We found weak evidence -8 and -7 seconds before delay onset for the model including the interaction between material and memory load ($1.5 < BF_{10} < 1.7$). Post-hoc tests for the memory load effect in the musical material revealed a positive to strong evidence for the memory load effect for the two tested time samples ($15 < BF_{10} < 24$). Pairwise post-hoc t-tests in the musical material revealed strong evidence for a difference between ML2 and ML3 ($19.3 < BF_{10} < 22.3$) with lower betas for the ML2 condition as compared to the ML3 condition. Finally, we found weak to positive evidence 1 to 4 seconds after delay onset for the model including the memory load effect ($1.7 < BF_{10} < 9.2$). Post-hoc tests averaged over materials for the memory load effect (not shown in Figure S6) revealed positive evidence in all tested time samples for a difference between ML1 and ML2 with lower betas for ML2 as compared to the ML1 condition. There was also weak evidence at 1 and 2 seconds for a difference between ML2 and ML3 ($1.1 < BF_{10} < 1.3$) with lower betas for the ML2 condition as compared to the ML3 condition.

For the SFG, we found weak to strong evidence -1 to 10 seconds around delay onset for the model including the material effect ($1 < BF_{10} < 74.9$) with lower betas for the musical material as compared to the verbal material.

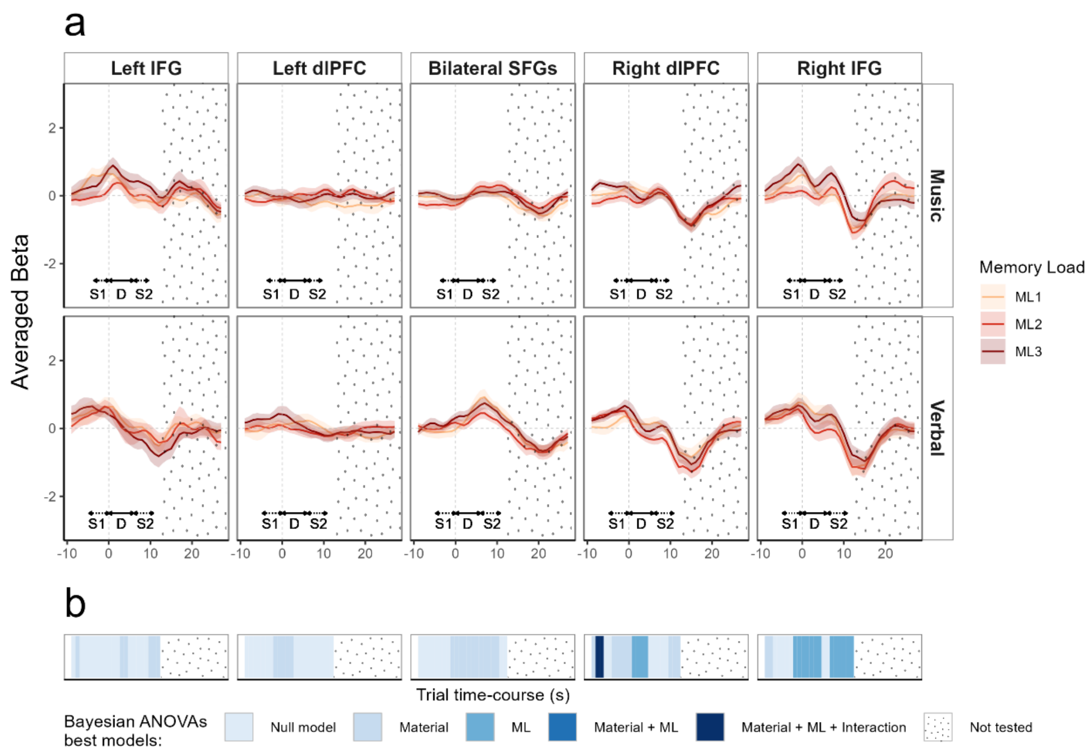

Figure S6: (a) Average beta (plain line) and standard error (shaded area) from the FIR deconvolution performed on HbR data for all participants (n=24), across five ROIs (left and right IFG, left and right dlPFC, bilateral SFGs), in a time window ranging from -9 to 27 seconds around delay onset (grey dotted vertical line), for the three memory load levels (ML1/ML2/ML3), for the musical material (top panel) and verbal material (bottom panel). For clarity purposes, S1, silent retention delay (D), and S2 durations are indicated with double-headed arrows, S1 and S2 arrows are dotted to indicate their variable duration according to the memory load. (b) Time-course representation of Bayesian analysis, each blue shade represents the best model as compared to the null model ($BF_{10} > 1$). No statistics were performed beyond 12 seconds after delay onset (end of S2 for the longest sequence + peak of the hemodynamic response, ~ 6s). Dotted areas in all panels represent portions of the signal that were not statistically analyzed.
